# Experimental Design in Polymer Chemistry—A Guide towards True Optimization of a RAFT Polymerization Using Design of Experiments (DoE)

**DOI:** 10.3390/polym13183147

**Published:** 2021-09-17

**Authors:** Tilman Eckert, Florian C. Klein, Piet Frieler, Oliver Thunich, Volker Abetz

**Affiliations:** 1 Helmholtz-Zentrum Hereon, Institute of Membrane Research, Max-Planck-Straße 1, 21502 Geesthacht, Germany; tilman.eckert@chemie.uni-hamburg.de; 2 Institute of Physical Chemistry, Universität Hamburg, Grindelallee 117, 20146 Hamburg, Germany; florian.klein-2@studium.uni-hamburg.de; 3 Statcon GmbH, Schulstraße 2, 37213 Witzenhausen, Germany; piet.frieler@statcon.de (P.F.); oliver.thunich@statcon.de (O.T.)

**Keywords:** design of experiment, reversible addition–fragmentation chain transfer, response surface methodology, prediction models, optimization

## Abstract

Despite the great potential of design of experiments (DoE) for efficiency and plannability in academic research, it remains a method predominantly used in industrial processes. From our perspective though, DoE additionally provides greater information gain than conventional experimentation approaches, even for more complex systems such as chemical reactions. Hence, this work presents a comprehensive DoE investigation on thermally initiated reversible addition–fragmentation chain transfer (RAFT) polymerization of methacrylamide (MAAm). To facilitate the adaptation of DoE for virtually every other polymerization, this work provides a step-by-step application guide emphasizing the biggest challenges along the way. Optimization of the RAFT system was achieved via response surface methodology utilizing a face-centered central composite design (FC-CCD). Highly accurate prediction models for the responses of monomer conversion, theoretical and apparent number averaged molecular weights, and dispersity are presented. The obtained equations not only facilitate thorough understanding of the observed system but also allow selection of synthetic targets for each individual response by prediction of the respective optimal factor settings. This work successfully demonstrates the great capability of DoE in academic research and aims to encourage fellow scientists to incorporate the technique into their repertoire of experimental strategies.

## 1. Introduction

As a powerful tool for efficient, reproducible, and predictable process optimization, DoE is firmly established in different fields of industry [1,2], process development [3], and engineering [4,5]. However, despite some promising recent works [6,7] and excellent surveying articles [8,9,10], DoE is still widely unknown in the realms of academic research. At least from our point of view, this might be a highly unfortunate omission as, if done right, DoE also provides superior understanding of the examined system and greater knowledge generation compared to conventional approaches to experimentation. On top of that, DoE generates so-called prediction models by appropriate fitting of the acquired experimental data, which accurately relate the experimentation parameters with a given observable result. These DoE-associated benefits not only emerge for examination of simpler cause and effect relationships but could also substantially advance workflows of more complex chemical reaction systems, such as RAFT polymerization.

RAFT is one of the most powerful and versatile controlled radical polymerization techniques [11] for the synthesis of complex multiblock architectures [12,13,14,15,16,17,18] as well as so-called “smart” materials, such as switchable filtration devices [19,20,21] or nanocarriers for biomedical applications [22,23,24]. Within this group of “smart” materials, especially upper critical solution temperature polymers, such as this work’s optimization target polymethacrylamide (PMAAm) [25,26], have received increasing attention [27,28,29,30].

During the RAFT process, many parameters influence the outcome of the polymerization and the quality of the final product. As RAFT is becoming increasingly relevant for industrial applications as well [31], optimization of RAFT processes remains a highly valuable task. Recently, various RAFT simulation studies have been published with promising results for kinetic computations [32,33] or specific challenges, such as transferring a RAFT polymerization into a microscale reactor [34]. However, simulations are usually limited by certain simplifications and require exact knowledge of the reaction mechanism. In the specific case of RAFT polymerization simulations, for example, the choice between the slow fragmentation model and intermediate radical termination model are still vividly discussed in literature [35,36,37]. DoE, on the other hand, does not rely on any theoretical assumptions regarding the examined system. In fact, correct application of DoE will always lead to accurate representation of the system as the prediction models are solely “fed” by experimental data (and thus also include all inevitable sources of error).

When applying RAFT to synthesize a polymer with tailored chain length, desired dispersity (oftentimes the lowest possible), and maximum chain end fidelity, polymer chemists are usually confronted with several challenges. The parameters, called factors in DoE terminology, impacting the results of a RAFT polymerization are manyfold; categorical factors, such as the type of solvent, RAFT agent, and initiator system, play a vital role. Luckily, for most types of monomers, thorough investigations regarding suitable solvents and RAFT agents have already been published [12,13,38,39,40]. Hence, it is rather common that the polymer chemist already knows all the participants of the reaction a priori. In most cases, the biggest challenge for a given goal of the polymerization lies in the optimal setting of all numeric factors. Depending on the kind of RAFT polymerization that is required, these factors can vary. Typically, these factors include reaction time and temperature, concentrations of reactants, and the ratios between them.

In order to optimize RAFT polymerization for any kind of synthetic goal, polymer scientists typically start with one polymerization, setting the factors based on their expertise. As it is highly probable that the result (e.g., the dispersity) is improvable, the impact of each factor can only be adequately investigated individually by varying solely one factor and repeating the experiment. Provided that the result changes, the investigated factor, for example, the temperature, will either be further adjusted or just kept at the favorable level. Now it is up to the expert to decide whether the result is satisfactory or if other factors need to be investigated individually as well. In the statistician community, this approach to experimentation is called the OFAT (one-factor-at-a-time) method and, expectably, does not enjoy the best reputation.

Clearly, in some cases, the OFAT method is useful or even without alternative. Contemplating the aforementioned scenario, however, certain critical questions seem appropriate:

1. Can we effectively spot the optimal setting (or level in DoE terminology) of each factor without innumerable experiments?
2. Can we just assume that an optimal factor level stays steadfast when another factor is varied?

**Figure 1:**
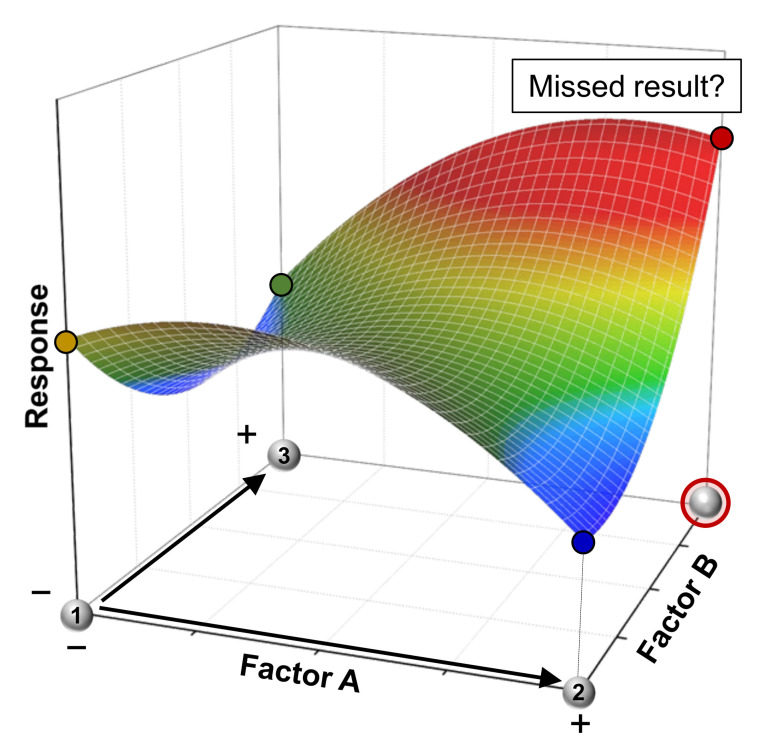
Strategic route of conventional OFAT experimentation. When interactions between factors are not revealed, a potential optimum might be missed.

Both questions can be answered with a simple “no”. The reason question 2 is negated is due to so-called factor interactions. In an example of a two-factor interaction AB, the effect of factor A on a quantifiable result, called response in the realms of DoE, depends on the factor level of factor B (and vice versa). This problem is illustrated in Figure 1, which displays an arbitrary response surface with three experiments at different factor settings. After starting the examination with a first experiment (factors are set to the levels A^−^ and B^−^, with the superscripts signifying high “+” or low “−” levels), factor A is investigated in typical OFAT fashion and increased to A^+^, resulting in a reduced response. If the response is maximized, the next experiment would likely be performed applying the factor levels A^−^ and B^+^ which, in this arbitrary example, again does not lead to the desired response enhancement. Presumably, not every scientist would go the extra mile and test the factor combination A^+^ and B^+^, an understandable choice that could lead to missing out on an unexpected result (provided the existence of a strong two-factor interaction AB).

At this point, it is obvious that the OFAT method runs against its limits. Fortunately, the statistical approach of experimental design provides solutions to all these problems. DoE takes a different approach to experimentation as it involves exploration of the whole experimental space. This way, all relevant interactions and nonlinear relations will be unveiled, and prediction models will be established that will ultimately disclose the optimal reaction conditions and save the experimenter valuable time and resources. This work will present a step-by-step guide for optimization of a thermally initiated RAFT solution polymerization of MAAm via DoE, highlighting the most important aspects along the way. We believe this work will not only be helpful for polymer chemists but also for interested scientists performing reactions in other disciplines of chemistry.

## 2. Materials and Methods

### 2.1. Materials

The RAFT agent CTCA (Sigma Aldrich, St. Louis, MO, USA, 95%), the thermal initiator ACVA, dimethyl formamide (DMF, VWR, Radnor, PA, USA, GPR Rectapure 99.5%), and acetone (Sigma Aldrich, St. Louis, MO, USA, 99.5%) were used as received. MAAm (Sigma Aldrich, St. Louis, MO, USA, 98%) was dried in vacuo for 24 h at room temperature and stored under nitrogen atmosphere. Ultrapure water (Milli-Q^®^ quality, resistivity >18.2 MΩ cm^−1^) was obtained from a Millipore Milli-Q^®^ water purification system and utilized as solvent for the RAFT polymerizations.

#### 2.1.1. RAFT Polymerization of MAAm

RAFT polymerizations were conducted in 12 mL screw-capped vials (Labsolute^®^) sealed with bored poly(propylene) caps and butyl/PTFE septa (Labsolute^®^, ND15, 1.6 mm). All polymerizations conducted within the scope of the FC-CCD were performed at a fixed mass of water of 3.000 g. In this fixed mass of Milli-Q^®^ water, the masses of MAAm, CTCA, and ACVA are, depending on the ratios ***R*_M_** and ***R*_I_**, exactly predefined to ensure the desired ***w*_s_** of the reaction solution. ***w*_s_** is the combined weight content MAAm, CTCA, and ACVA with respect to the whole reaction solution (precluding internal standard DMF). A typical thermal RAFT polymerization for all factors (***T***, ***t***, ***R*_M_**, ***R*_I_**, and ***w*_s_**) at their center levels was conducted as follows. MAAm (533 mg, 6.26 mmol, ***R*****_M_** = 350) and CTCA (5.6 mg, 18 µmol) were dissolved in Milli-Q^®^ water. The required mass of ACVA (31 µg, 1.12 µmol, ***R*_I_** = 0.0625) was added using an Eppendorf Multipette^®^ E3, which transferred the appropriate volume from an ACVA solution (10 mg mL^−1^) in DMF into the reaction mixture. As the DMF serves as internal standard for monomer conversion determination via ^1^H NMR spectroscopy, more DMF was added to employ a DMF concentration of 5 wt % (in terms of total mass of the final reaction mixture). Afterwards, the mixture was homogenized under vigorous stirring, and a small sample was taken for NMR referencing. As already observed in previous works, neither the MAAm nor the DMF signal are affected by subsequent N_2_ bubbling [25]. The homogenized reaction solution was purged by N_2_ bubbling for 10 min. The solution was stirred (600 rpm) at ***T*** = 80 °C for ***t*** = 260 min and quenched by rapid cooling to 0 °C and exposure to air. Another sample was taken for NMR analysis, and the solution was precipitated by dropwise addition into 60 mL of ice-cold acetone. The precipitate was filtrated and dried in vacuo for 24 h at room temperature. PMAAm was obtained as a yellowish powder (***p*** = 42.7%. ${\bar{M}}_{n,\mathrm{th}}$ = 12.8 kDa, ${\bar{M}}_{n,\mathrm{app}}$ = 6.2 kDa, ***Đ*** = 1.28). Experimental design, ANOVA, model prediction, and diagnostics were performed using the software DesignExpert^®^ V12.

### 2.2. Analytics

#### 2.2.1. Nuclear Magnetic Resonance Spectroscopy

^1^H NMR measurements were performed with a Bruker^®^ AVANCE III HD 400 MHz spectrometer. Spectra were recorded at a temperature of 300 K with 64 scans per spectrum and a delay of three seconds. Deuterium oxide (D_2_O) was used as solvent, and the residual solvent peak was set at 4.79 ppm and used as reference. The sample concentration was approximately 10 mg mL^−1^ for all measurements. In order to determine the monomer conversion of a typical RAFT polymerization of MAAm, DMF was added and the DMF/monomer integral ratios before the polymerization and at a given polymerization time were compared (see Figure S1 as well as Equations S1 and S2 in the Supporting Information).

#### 2.2.2. Size Exclusion Chromatography

Size exclusion chromatography (SEC) experiments were conducted on a PSS^®^ Agilent Technologies 1260 Infinity system utilizing a SUPREMA^®^ column system consisting of a precolumn (8 mm × 50 mm, particle size: 5 µm) and three analytical columns (column 1: 8 mm × 300 mm, particle size of 5 µm, mesh size of 1000 Å; column 2: 8 mm × 300 mm, particle size of 5 µm, mesh size of 1000 Å; and column 3: 8 mm × 300 mm, particle size of 5 µm, mesh size of 30 Å). All columns were always kept at a constant temperature of 50 °C. A 0.1 M NaNO_3_ aqueous (Milli-Q^®^ quality) solution with an added 0.05 wt % NaN_3_ served as eluent, while concentrations of PMAAm varied between 1 and 2 mg mL^−1^. The measurements were conducted at a flow rate of 1 mL min^−1^ by applying an isocratic PSS^®^ SECcurity pump and ethylene glycol (20 µL per 100 mL of solvent) as internal standard. Poly(ethylene glycol) was used as reference, and the PSS^®^ SECcurity differential refractometer detector was operated at 50 °C. Subsequent data analysis was performed with the software WinGPC UniChrom^®^ V8.10.

## 3. Results and Discussion

### 3.1. Screening: Finding the Significant Factors

**Figure 2:**
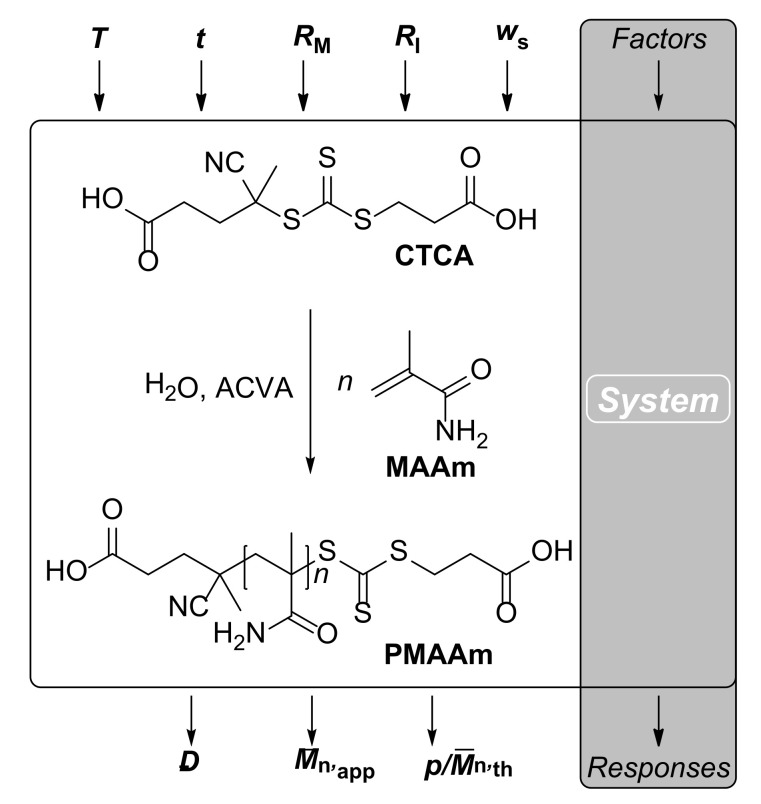
Polymerization of MAAm in aqueous solution utilizing CTCA as RAFT agent and ACVA as initiator. The responses of the polymerization system dispersity ***Đ***, apparent number averaged molecular weight ${\bar{M}}_{n,\mathrm{app}}$, and monomer conversion ***p***(as well as the theoretical number averaged molecular weight ${\bar{M}}_{n,\mathrm{th}}$, which is proportional to ***p***) are influenced by the factors of reaction temperature ***T***, reaction time ***t***, the initial concentration ratios of [MAAm]/[CTCA] ***R*_M_** and [ACVA]/[CTCA] ***R*_I_**, and the total solid content ***w*_s_**.

A schematic display of the RAFT polymerization of MAAm (the examined system) is given in Figure 2. The polymerization of MAAm was performed in aqueous solution with the use of the thermal azo initiator 4,4′-azobis(4-cyanovaleric acid) (ACVA) and RAFT agent 4-(((2-carboxyethyl)thio)carbonothioyl)thio-4-cyanopentanoic acid (CTCA).

Only three factors are required for the unequivocal description of the RAFT reaction solution: the ratios ***R*_M_** and ***R*_I_**, which represent the initial concentration ratios of [MAAm]/[CTCA] and [ACVA]/[CTCA], respectively, as well as the total solid content ***w*_s_**, which is the combined mass content of MAAm, CTCA, and ACVA of the reaction solution precluding *N*,*N*-dimethyl formamide (DMF, see Section 2.1.1.). At a designated solvent mass of H_2_O, the combination of these three factors unambiguously predefines the reaction solution. The influence of ***w*_s_** on the reactant solutions is rather straightforward as it determines the total mass to be occupied by MAAm, CTCA, and ACVA. The interplay of ***R*_M_** and ***R*_I_**, however, is a bit more complicated. While [MAAm] and [CTCA] stay largely unaffected by ***R*_I_** (as the weight content of ACVA will always be negligibly low), [ACVA] strongly depends on both ***R*_M_** and ***R*_I_** (high initiator concentrations are obtained at low ***R*_M_** values and high ***R*_I_** values).

Before performing any DoE, it is generally mandatory to specify the responses of interest. For example, when performing a RAFT homopolymerization, scientists are typically interested in three polymer attributes: the molecular weight, the molecular weight distribution (represented by the dispersity ***Đ***), and the chain end fidelity. Unfortunately though, the latter quantity is very difficult to obtain analytically and in a reliable and precise fashion due to various imponderabilities. Therefore, the responses monitored within the scope of our experimental designs were ***Đ****,* the monomer conversion ***p***, and the apparent number averaged molecular weight ${\bar{M}}_{n,\mathrm{app}}$ (obtained from size exclusion chromatography (SEC) using poly(ethylene glycol) standards). An additional response is the theoretical number averaged molecular weight ${\bar{M}}_{n,\mathrm{th}}$ which is proportional to ***p*** (and determined via ^1^H NMR spectroscopy, as shown in Section S1 and Figure S1, Supporting Information).

As any RAFT expert knows, apart from ***R*_M_**, ***R*_I_**, and ***w*_s_**, two additional factors clearly possess significant influence on the outcome of ***p***, ${\bar{M}}_{n,\mathrm{th}}$, ***Đ***, and ${\bar{M}}_{n,\mathrm{app}}$, namely the reaction temperature ***T*** and the reaction time ***t***. Fortunately, DoE is not only apt to evaluate the obviously significant factors but can also classify the more unimposing parameters. The step of testing factors to extract the significant few (i.e., factors exerting a significant effect on at least one of the system’s responses) from the insignificant many is commonly called screening. The screening is usually built of a two-level design with low amounts of experiments (which are called runs within the scope of experimental design), only allowing for modeling of linear relationships. Consequently, screenings mostly serve the qualitative purpose of the distinction between significant and insignificant factors. Deeper understanding and adequate mathematical predictions of more complex, nonlinear relationships will only be achieved with subsequent more sophisticated designs, such as the so-called response surface methodology (RSM, see Section 3.3).

Typically, at the beginning of any screening process, a group of experts will gather to brainstorm and think of all factors potentially impacting at least one response and subsequently examine them within a low-run design. In some cases though, it might be redundant to inspect clearly significant factors; experts often already know some of the factors that need to be examined in the ensuing RSM design. Thus, an efficient DoE specialist can save on the number of required screening runs by setting all unequivocally significant factors at a reasonable level and only testing the remaining, less predictable ones. In our case, for example, ***T***, ***t***, ***R*_M_**, ***R*_I_**, and ***w*_s_** were excluded from the screening as we already knew of their significance. It must be stated that screening designs can theoretically suffice for precise modeling of the most complex systems if only linear relations are investigated. However, as soon as a single factor has a nonlinear effect on any response, a design with the capability to examine higher order model terms is required. Because, for example, ***p*** will most certainly not correlate linearly with ***t*** for any kind of chain growth polymerization, later investigation of the RAFT polymerization of MAAm via an RSM design was imperative (see Section 3.3).

Thinking of factors potentially influencing ***p***, ${\bar{M}}_{n,\mathrm{th}}$, ***Đ***, and ${\bar{M}}_{n,\mathrm{app}}$, more than the obvious five come to mind. Prior to a thermally initiated RAFT polymerization, a designated solvent mass (defined by the mass of the applied solvent H_2_O), stirring rate, N_2_ purging time, and concentration of internal standard (DMF in our case) need to be selected. Naturally, all these factors can certainly drastically influence polymerization. In small scales and within reasonable factor ranges though, it is commonly assumed and rightfully so that their impact is negligible. A pleasant feature of DoE is that these assumptions of negligibility can easily be confirmed or in other cases refuted with statistical security. As expected, after sufficient N_2_ purging and within low scaled ranges of H_2_O solvent mass (1−5 g), stirring speed (400–800 rpm), and DMF concentration (2−5 wt %), none of these factors turned out to be significant (for detailed display of the conducted screening see Tables S1 and S2, Supporting Information).

As larger numbers of factors included into the subsequent RSM design equal markedly higher experimental and monetary effort, proving factors to be insignificant in the screening is usually a rather welcome finding. In our particular case, this allowed for a complacent conclusion, namely execution of an RSM design with only ***T***, ***t***, ***R*_M_**, ***R*_I_**, and ***w*_s_** (which we ex ante knew to have significant influence) will fully suffice for thorough comprehension of the RAFT polymerization of MAAm.

### 3.2. Setting Factor Levels and Choosing the Design

Setting the factor levels is by no means an easy task. In fact, this step can be one of the biggest challenges of DoE. While wide factor ranges grant information over a larger experimental space, optimal prediction accuracy (and prevention of factor correlations) will only be achieved when a quantifiable value for each response is obtainable for every single run, even for the most “extreme” set of factor combinations. In our case, the lowermost response for ***p*** is expected at the factor levels ***R*_M_^+^**, ***R*_I_^−^**, and ***w*_s_^−^** as this combination leads to minimal ACVA concentration. Coupled with the low levels of ***T*** and ***t***, the lowest conversion of the whole RSM design will most likely be obtained. As a result, parts of the polymer’s molecular weight distribution might fall outside of the SEC column permeation limit, leading to a corrupt or even missing response. To sum things up, finding a sweet spot between a large experimental space (caused by widely spread factor levels) and assuring run feasibility is essential. Hence, before starting elaborate experimental designs, a prudent scientist will perform appropriate preliminary tests.

An additional and essential element of RSM that is potentially able to impact the design’s feasibility is the design itself. Depending on the design’s geometry, certain factor combinations are required or evaded. There is a wide range of viable RSM designs with various geometries, each one offering certain assets as well as challenges [41]. The RSM of our choice was a FC-CCD, which allows for great prediction accuracy whilst examining each factor at three levels only. The experimental geometry is shown in the Supporting Information (Figure S2).

Table 1 lists the low (−) and high (+) as well as the center (0) level for all five factors examined in the FC-CCD. The levels were carefully selected after appropriate preliminary testing with regard to the design geometry and SEC permeation limits. The prediction models for each response will be valid within these exact level ranges; extrapolation is error-prone.

Being aware of all significant factors, a well-suited combination of factor levels, and the design geometry, the experimental design can be constructed (see Table S3, Supporting Information). After completion of all runs and generation of the respective response data, the prediction models can be generated by polynomial regression and revised via analysis of variance (ANOVA) and appropriate model diagnostics tools.

### 3.3. Response Surface Methodology: Generation and Interpretation of Prediction Models

Depending on the applied design, prediction models with certain polynomials can be established. The FC-CCD allows for generation of a quadratic model, while higher ordered terms, such as $T^{3}$, will be confounded (i.e., unambiguous estimation is impossible). The challenge of finding out which polynomial terms are significant and thus should be included in the prediction model is mastered by an ANOVA of the regression model as well as evaluation of so-called goodness-of-fit measures (see Supporting Information), which will typically be computed by appropriate statistical software (we used DesignExpert^®^).

The resulting coded prediction models for ***p***, ${\bar{M}}_{n,\mathrm{app}}$, and ***Đ*** are listed in Equations (1)–(3) (respective ANOVA tables and relevant fit statistics are tabulated in Tables S4–S11 and model diagnostics are displayed in Figures S3–S6 in the Supporting Information). Due to the proportionality of ***p*** and ${\bar{M}}_{n,\mathrm{th}}$, both models are highly similar (thus the model of ${\bar{M}}_{n,\mathrm{th}}$ is shown in Table S7 of the Supporting Information. The models are “coded”, i.e., the actual factor ranges are converted to −1 and +1. In this form, the intercept (the first term of each equation) signifies the average of all responses. Moreover, the coefficients *β* are dimensionless, thereby granting straightforward assessment of each term’s effect size. If, for example, all factors are set to their center level (0), the model for ***p*** predicts a conversion of 42.3%. Changing the temperature to the low level of 75 °C (coded level: −1), will, according to the prediction model, result in a conversion of 37.1% (also mind the $T^{2}$ term). In this manner, the equations allow for comparison of factor effects (within the observed factor level ranges) as well as cause interpretation of each individual model term.

(1) $$p/\%=42.3+3.10\;T+12.7\;t-6.31\;R_{M}+9.59\;R_{I}+4.78\;w_{s}-2.06\;T\;t+0.719\;T\;R_{I}+0.275\;T\;w_{s}-1.74\;t\;R_{M}+1.28\;t\;R_{I}+1.52\;t\;w_{s}-1.09\;R_{M}\;R_{I}-2.08\;T^{2}-4.28\;t^{2}+2.62\;R_{M}^{2}-2.88\;R_{I}^{2}-1.17\;T\;t\;R_{I}-0.725\;T\;t\;w_{s}$$

(2) $${\bar{M}}_{n,\mathrm{app}}/\mathrm{kDa}=6.23+0.259\;T+1.25\;t-1.06\;R_{M}+0.927\;R_{I}+0.488\;w_{s}-0.259\;T\;t+0.209\;T\;R_{M}+0.053\;T\;w_{s}+0.453\;tR_{M}+0.109\;t\;R_{I}+0.322\;t\;w_{s}+0.278\;R_{M}\;R_{I}+0.278\;R_{M}\;w_{s}+0.047\;R_{I}\;w_{s}-0.366\;T^{2}-0.766\;t^{2}-0.134\;T\;t\;w_{s}-0.303\;t\;R_{I}\;$$

(3) $$Đ/a.u.=1.26+0.04\;T+0.0194\;t+0.0135\;R_{M}-0.0194\;R_{I}-0.0297\;w_{s}+0.025\;T\;t-0.0144\;T\;R_{I}-0.0025\;T\;w_{s}+0.0188\;t\;w_{s}+0.0131\;R_{I}\;w_{s}+0.0307\;t^{2}+0.0212\;t\;R_{I}\;$$

**Figure 3:**
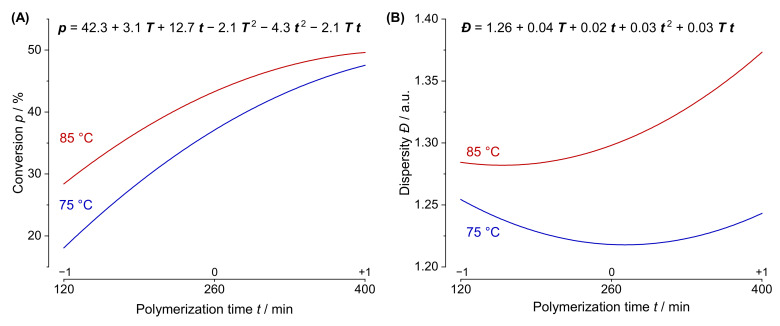
Illustration of two temperature–time interaction plots. The responses (***p*** in graph (**A**) and ***Đ*** in graph (**B**)) are plotted against ***t*** for the temperatures of 75 °C (blue line) and 85 °C (red line), whilst the factors ***R*_M_**, ***R*_I_**, and ***w*_s_** are set to their center (0) level. The respective coded prediction models are additionally displayed.

Despite being rather bulky equations, the prediction models offer comprehensive and valuable information. For any set of factors (i.e., reaction parameters) within the given experimental space, all system responses of the polymerization can be forecast. At this point though, it is evident that prediction models offer more than the mere forecast of responses at given factor combinations. In fact, the models provide great potential for academic purposes and qualitative understanding of the examined system as they conclusively reveal the impact of each model term. This grand avail becomes particularly obvious when looking at factor interactions. To demonstrate this, Figure 3 illustrates the ***T t***-interactions for of the ***p*** and ***Đ*** prediction model. Within this illustration, all factors except ***T*** and ***t*** are set to their center (0) level. In both graphs, the respective predicted responses are plotted versus ***t*** for the temperatures of 75 °C (blue line) and 85 °C (red line); the respective prediction models are also displayed. The two plots in graph A depict two typical saturation functions. The model coefficients of temperature *β_T_* and time *β_t_* are greater than 0 (*β_T_* = 3.10 and *β_t_* = 12.7), indicating that ***p*** will increase along with both factors, which is an expected result. With progressing ***t***, more monomer is consumed, while elevated ***T*** values facilitate quicker reaction rates according to the Arrhenius law. The interpretation of the quadratic coefficients *β_T2_* and *β_t2_* (−2.08 and −4.28, respectively) is also straightforward. As both are negative, the slope of the conversion will recede (whether plotted against ***T*** or ***t***). This phenomenon can be attributed to the continuous depletion of monomer, which is directly proportional to the rate of polymerization [42].

In spite of this, actual and often nontrivial insights are usually acquired by interpretation of the interactions. As the interaction coefficient *β_T t_* ≠ 0, the effect of ***t*** on the monomer conversion will vary for different values of ***T*** and vice versa, or to put it in other words, the prediction plots for the ***t*** dependence at different levels of ***T*** will be nonparallel. Within the examined time range, the average polymerization rate (i.e., the slope of ***p*** against ***t***) appears to be higher at 75 °C, which is a rather abnormal observation. Apparently, the polymerization rate at 85 °C is suppressed (compared to the one at 75 °C). This might be due to the slightly lower monomer concentration (at 120 min, ***p*** is already higher at 85 °C). Yet, this result is much more plausibly explained by an insufficient radical generation. As around 98% of the initial [ACVA] has decomposed after only 260 min at 85 °C in water, the concentration of growing active species may be significantly reduced, especially towards the later stages of the polymerization (for calculation of initiator decomposition see chapter 4 and Equations S4 and S5, Supporting Information) [43].

Part B of Figure 3 displays the ***Đ*** prediction plots versus ***t*** at the two temperatures of 75 °C (blue line) and 85 °C (red line) as well as the respective coded prediction model (***R*_M_**, ***R*_I_**, and ***w*_s_** are set to their center level). Overall, higher ***Đ*** values are obtained at higher temperatures (*β_T_* > 0). At 75 °C, ***Đ*** initially decreases with polymerization time, which we can assume correlates with higher average chain lengths in well-controlled radical polymerizations (CRP). This result comes as no surprise as CRPs follow the Poisson distribution, leading to lower dispersity with growing degree of polymerization [44]. After the initial descent, ***Đ*** actually passes a minimum before increasing towards the later stages of polymerization (*β_T2_* > 0). This might be due to the increased likelihood of irreversible termination reactions or hint towards thermal decomposition of the trithiocarbonate over the course of polymerization. Thermal decomposition of RAFT agents is a well-reported phenomenon known to broaden the resulting polymer’s molecular weight distribution [45,46], with trithiocarbonates attached to acrylates being particularly labile [47]. The increase of ***Đ*** is even more pronounced (and starts earlier) at higher temperatures (*β_T t_* > 0), strengthening this argument.

Interpretation of the two interaction plots of Figure 3 is just an example of the prospects of DoE when it comes to knowledge generation and true understanding of the examined system. Information that would potentially be lost via conventional OFAT experimentation will be revealed in a reliable and plannable fashion. Alongside these academic prospects, it can be easy to forget the other great benefit of the prediction models, namely the great capability when it comes to tailored and targeted syntheses. This virtue will be presented in the following section.

### 3.4. True Optimization

With the help of the prediction models, scientists are able to find the optimal factor settings for various different synthetic goals. Such goals might be to minimize or maximize a response, to stay within an adjustable range, or to achieve a certain response target. Remarkably, these exact goals can also be defined for the factors themselves. Deploying a scientist’s demands, appropriate statistical software will compute the respective optimal factor settings (this is typically achieved through application of a so-called desirability algorithm) [48].

At this point, a RAFT specialist may notice another highly pleasant feature that the prediction models will provide optimized reaction conditions (i.e., factor combinations) for a wide range of selectable ${\bar{M}}_{n,\mathrm{th}}$ targets (in our case between 2.1 and 25.1 kDa). In conventional OFAT experimentation, ${\bar{M}}_{n,\mathrm{th}}$ targets will usually be achieved by picking a fixed value of ***R*_M_** and polymerizing up to the corresponding monomer conversion (see Equation (S2), Supporting Information). The resulting ***Đ*** value (or other potential quantities of interest) will then be optimized by modification of parameters such as ***T***, ***w*_s_**, or the initiator concentration. For targeting a certain chain length though, a full kinetic study will be required for each modification as the polymerization rate will most likely be affected. In other words, the achievement of a comparably sophisticated, comprehensive and reliable optimization for even just a single ${\bar{M}}_{n,\mathrm{th}}$ target is nearly unobtainable via conventional OFAT experimentation.

### 3.5. Model Validation

One purpose of this work is to demonstrate the perks of experimental design, even in relatively complex reactions such as CRPs. Literal optimization, however, was not aspired as the “optimal” polymer always depends on the later application purpose. In many cases though, DoE is deployed for actual optimization of certain specific responses. As soon as the optimal factor settings for a given goal are provided by the models, despite full confidence in statistics, it is strongly advised to empirically validate the prediction experimentally.

As no actual optimization target existed within the scope of this work, three arbitrarily picked polymerization goals were defined. Herein, three relatively diverse ${\bar{M}}_{n,\mathrm{th}}$ values with specific (or minimal) ***Đ*** values were targeted. In Table 2, the predicted responses for the three arbitrarily chosen polymerizations are listed, with the targets highlighted in bold letters (respective factor settings are shown in Table S12, Supporting Information).

**Figure 4:**
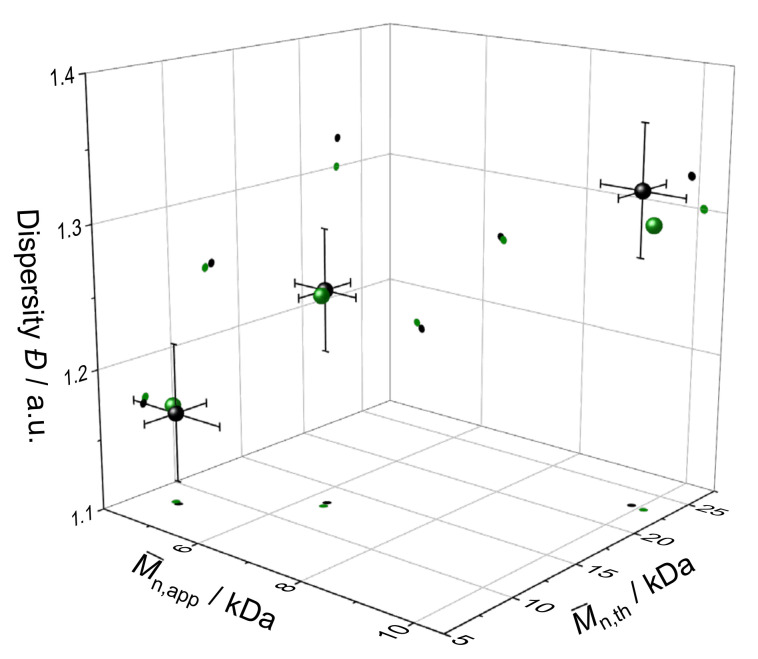
Predicted (black spheres) and observed (green spheres) responses of ${\;M\;¯}_{n,\mathrm{th}}$, ***Đ***, and ${\bar{M}}_{n,\mathrm{app}}$ as well as the 95% PIs for the three arbitrarily picked polymerization goals. Value projection with respective coloring on the response planes is added as a guide to the eye.

The prediction models were confirmed empirically by performing additional validation runs and comparison of observed and predicted responses. Figure 4 illustrates the predicted responses (black spheres) for ${\bar{M}}_{n,\mathrm{th}}$, ***Đ***, and ${\bar{M}}_{n,\mathrm{app}}$ (***p*** is left out due to redundancy with ${\bar{M}}_{n,\mathrm{th}}$) and the observed responses (green spheres) as well as the so-called 95% prediction intervals (PIs) of the three arbitrarily picked polymerization goals. A PI is an estimate of an interval in which 95% of the observed responses of individual repetitions should fall based on the examined system’s systematic error. By definition, the model is validated empirically if the observed response falls within the 95% PI.

Clearly, the prediction models predicted the experimental findings with exceptional accuracy (comprehensive validation data is given in Table S13, Supporting Information). The model is confirmed.

## 4. Conclusions

This work may serve as an introductory example for scientists who have been unaware of the great potential of experimental design in their fields of academic research. DoE was shown to be eminently well suited for the complex reaction system of RAFT polymerization. Highly accurate prediction models offering greater and statistically credible understanding of the examined system were generated, and a step-by-step presentation of the DOE-associated workflow was provided. Within appropriate scales of solvent mass, three factors (***R*_M_**, ***R*_I_**, and ***w*_s_**) sufficed for unambiguous definition of the reaction solution. Additional incorporation of factors ***T*** and ***t*** facilitated a comprehensive mathematic portrait of the whole reaction system of thermally initiated RAFT solution polymerization of MAAm. Within this context, it was shown that DoE not only provides routes towards potential “sweet spots” of the examined system but also offers grand potential for generation of knowledge and thorough qualitative understanding. In particular, the detection and interpretation of two- or even three-factor interactions, which would most likely stay hidden without utilization of DoE, reveals decisive advantages over conventional OFAT experimentation.

Therefore, we think that not only RAFT experts but also scientists of other disciplines might find it useful to incorporate DoE into their research. However, when doing so, it is important to state that for every new specific system with a certain specific goal, appropriate adaptations to our proposed route might be necessary. Obviously, a system other than a thermally initiated RAFT polymerization will require different factors, new setting of factor levels, and perhaps even utilization of a better suited design geometry than an FC-CCD. Here, the so-called computer-generated optimal designs have especially received attention due to their great flexibility and often optimal compromise of prediction accuracy and experimental effort [49,50,51].

Despite these hurdles, we want to encourage scientists to integrate DoE into their repertoire of experimental strategies and employ it routinely into their workflows. At first, some help by excellent textbooks [52,53] or cooperative statisticians may be necessary. In the long run, however, DoE can be highly beneficial and just might facilitate an unexpected breakthrough.

## Figures and Tables

**Table 1:** Low (−), center (0) and high (+) level for each factor examined in the FC-CCD.

	*T*/°C	*t*/min	*R*_M_/a.u.	*R*_I_/a.u.	*w*_s_/a.u.
−	75	120	200	0.025	10.0
0	80	260	350	0.0625	15.0
+	85	400	500	0.1	20.0

**Table 2:** Predicted responses of arbitrarily chosen polymerizations for empiric validation of the prediction models. The polymerization targets are highlighted in bold letters.

Goal	${\bar{M}}_{n,\mathrm{th}}{\;}^{a}/\mathrm{kDa}$	*p*^a^/%	${\bar{M}}_{n,\mathrm{app}}{\;}^{b}/\mathrm{kDa}$	*Đ*^b^/a.u.
1	**8.0**	45.2	4.70	**minimize**
2	**13.1**	57.5	6.35	**1.25**
3	**23.0**	58.8	9.87	**1.32**

^a^ Determined via ^1^H NMR spectroscopy and referencing to DMF. ^b^ Measured by SEC at 50 °C in an 0.1 M NaNO_3_ aqueous (Milli-Q^®^ quality) solution with an added 0.05 wt % NaN_3_ and calibration with poly(ethylene glycol).

## Data Availability

The data presented in this study are available on request from the corresponding author.

## Supplementary Materials

The following are available online at https://www.mdpi.com/article/10.3390/polym13183147/s1, Figure S1: Relevant parts of two ^1^H NMR spectra of a MAAm homopolymerization, Figure S2: Design geometry of an arbitrary three-factor FC-CCD, Figure S3: Diagnostic plots for the prediction model of ***p***, Figure S4: Diagnostic plots for the prediction model of ${\bar{M}}_{n,\mathrm{th}}$, Figure S5: Diagnostic plots for the prediction model of ${\bar{M}}_{n,\mathrm{app}}$, Figure S6: Diagnostic plots for the prediction model of ***Đ***, Table S1: Standard order experimental design of the screening, Table S2: ANOVA tables for the responses ***p****,* ${\bar{M}}_{n,\mathrm{th}}$, ***Đ***, and ${\bar{M}}_{n,\mathrm{app}}$**,** Table S3: Standard order experimental design of the FC-CCD, Table S4: ANOVA table for the response ***p***, Table S5: Coded prediction model of ***p***, Table S6: ANOVA table for the response ${\bar{M}}_{n,\mathrm{th}}$, Table S7: Coded prediction model of ${\bar{M}}_{n,\mathrm{th}}$, Table S8: ANOVA table for the response ${\bar{M}}_{n,\mathrm{app}}$, Table S9: Coded prediction model of ${\bar{M}}_{n,\mathrm{app}}$, Table S10: ANOVA table for the response ***Đ***, Table S11: Coded prediction model of ***Đ***, Table S12: Listing of the paradigmatic polymerization goals and their respective optimal factor settings, Table S13: Predicted and observed responses as well as the 95% PIs of the three paradigmatic polymerization goals.
