# Supplementary material for: Experimental Design in Polymer Chemistry—A Guide towards True Optimization of a RAFT Polymerization Using Design of Experiments (DoE)
Source: Polymers (Basel). 2021 Sep 17;13(18):3147. doi: 10.3390/polym13183147 (PMC8468855; doi:10.3390/polym13183147)
Supplement: Supplementary file 1 [file polymers-13-03147-s001.zip › polymers-1374573-supplementary.pdf]

Experimental Design in Polymer Chemistry – A Guide Towards True Optimization of a RAFT Polymerization using Design of Experiments (DoE)

Tilman Eckert,[1,2] Florian C. Klein,[2] Piet Frieler,[3] Oliver Thunich,[3] Volker Abetz[1,2]

[1]Helmholtz-Zentrum Hereon, Institute of Membrane Research, Max-Planck-Straße 1, 21502 Geesthacht, Germany.
Correspondence to: Volker Abetz (E-mail: volker.abetz@hereon.de)
[2]Institute of Physical Chemistry, Universität Hamburg, Grindelallee 117, 20146 Hamburg, Germany
[3]Statcon GmbH, Schulstraße 2, 37213 Witzenhausen, Germany

1 NMR Analysis

1.1 Determination of the MAAm conversion

In the following, the relevant parts of two ^{1}H NMR spectra of a MAAm homopolymerization are illustrated (see Figure S1). The relevant NMR signals for the determination of the monomer conversion are assigned to their associated protons. The top spectrum depicts the reaction solution after a given polymerization time (a certain amount of monomer was converted into polymer as can be seen by the broad polymer signals at approximately 2.25–0.90 ppm). The lower spectrum derives from the reaction solution before the start of the polymerization by light irradiation (as indicated by “0” in the signal indices). When setting the highlighted DMF signal integral to 1, the relevant monomer signal integrals can be compared. The monomer conversion p was determined via Equation S1:

$$p = 1 - \frac{I_{\mathbf{A'}} + I_{\mathbf{B'}}}{I_{\mathbf{A_0}} + I_{\mathbf{B_0}}}. \quad \text{(S1)}$$

Having determined the monomer conversion, now the theoretical number average molecular weight $\overline{M}_{\mathrm{n,th}}$ could be computed:

$$\overline{M}_{\mathrm{n,th}} = \frac{p\,[\mathrm{MAAm}]_0 M_{\mathrm{MAAm}}}{[\mathrm{CTCA}]_0} + M_{\mathrm{CTCA}}\,. \quad \text{(S2)}$$

In Equation S2, M_{M} and M_{CTCA} are the molecular weights of the MAAm and the RAFT agent, respectively, while $[\mathrm{MAAm}]_0$ and $[\mathrm{CTCA}]_0$ are their concentrations in the initial reaction solution.

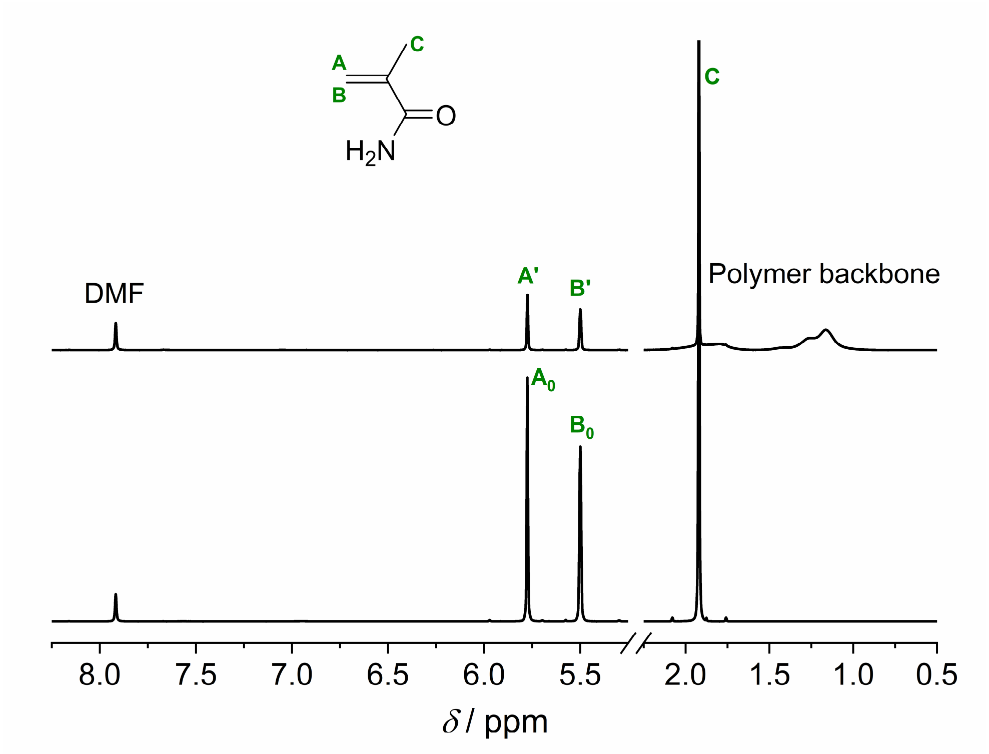

Figure S1: Relevant parts of two ^{1}H NMR spectra of a MAAm homopolymerization for the determination of the monomer conversion as well as assignment of the relevant signals.

2 Screening

In the screening, the influence of the reaction volume (defined by the mass of the applied solvent H_2O m_{H_2O}), concentration of internal standard w_{DMF}, the stirring speed r and the N_2-purging time t_{N_2} on the responses p, $\overline{M}_{n,th}$, $Đ$ and $\overline{M}_{n,app}$ was investigated.
A fractional factorial two-level design (2^{4-1} runs; resolution IV) with 4 additional center points was conducted. The experimental design with the actual factor settings and the measured responses is given in Table S1. For purposes of better overview the runs are listed in standard order. However, to avoid lurking time-related errors, the practical order of runs was randomized. The twelve resulting RAFT polymerizations of MAAm were each conducted at equal reaction conditions ($T = 80$ °C, $t = 260$ min, $R_M = 350$, $R_I = 0.0625$, $w_s = 15$wt%).

Table S1: Standard order experimental design of the screening. Display of the actual factor values as well as the observed responses.

Run	m_{H_2O} / g	w_{DMF} / wt%	r / rpm	t_{N_2} / min	p^a / %	$\overline{M}_{n,th}{}^a$ / kDa	$Đ^b$ / a.u.	$\overline{M}_{n,app}{}^b$ / kDa
**1**	1.00	2.0	400	5	45.2	11.9	1.20	6.4
**2**	5.00	2.0	400	15	46.7	12.1	1.16	6.4
**3**	1.00	5.0	400	15	40.2	10.5	1.17	5.6
**4**	5.00	5.0	400	5	43.3	11.3	1.17	6.4
**5**	1.00	2.0	800	15	42.3	11.0	1.18	5.7
**6**	5.00	2.0	800	5	41	10.8	1.15	7.4
**7**	1.00	5.0	800	5	41.4	10.4	1.20	6.6
**8**	5.00	5.0	800	15	44.3	11.6	1.19	6.1
**9**	3.00	3.5	600	10	44.9	11.8	1.2	6.7
**10**	3.00	3.5	600	10	44	11.6	1.18	6
**11**	3.00	3.5	600	10	42.7	11.4	1.15	6.8
**12**	3.00	3.5	600	10	46.8	12.3	1.16	6.1

[a] Determined via ^{1}H NMR spectroscopy and referencing to DMF. [b] Measured by SEC at 50 °C in an 0.1 M $NaNO_3$ aqueous (Milli-Q® quality) solution with an added 0.05wt% NaN_3 and calibration with poly(ethylene glycol).

The analysis of variance (ANOVA) tables for all four responses are listed in Table S2. Despite the fact that none of the three individual ANOVAs included any factor into their models, the respective lack of fit is not significant (p-value > 0.05 in each ANOVA). This means all four tested factors are insignificant as they have no effect on the observed responses within their examined factor ranges. Also, the p-value of the curvature is higher than 0.05 in each of the three ANOVAs indicating that no significant non-linearity was observed.

Table S2: ANOVA tables for the responses *p*, $\overline{M}_{n,th}$, *Đ* and $\overline{M}_{n,app}$.

Source	Sum of Squares	df	Mean Square	F-value	p-value
**Model of *p***	0.0000	0			
Curvature	6.41	1	6.41	1.45	0.2557
Residual	44.08	10	4.41		
Lack of Fit	35.18	7	5.03	1.69	0.3593
Pure Error	8.90	3	2.97		
Cor Total	50.49	11			
**Model of $\overline{M}_{n,th}$**	0.0000	0			
Curvature	0.8817	1	0.8817	2.71	0.1304
Residual	3.25	10	0.3248		
Lack of Fit	2.80	7	0.4000	2.68	0.2246
Pure Error	0.4475	3	0.1492		
Cor Total	4.13	11			
**Model of *Đ***	0.0000	0			
Curvature	0.0001	1	0.0001	0.1743	0.6851
Residual	0.0038	10	0.0004		
Lack of Fit	0.0023	7	0.0003	0.6828	0.6957
Pure Error	0.0015	3	0.0005		
Cor Total	0.0039	11			
**Model of $\overline{M}_{n,app}$**					
Curvature	0.6337	1	0.6337	3.02	0.1127
Residual	2.10	10	0.2096		
Lack of Fit	1.15	7	0.1641	0.5196	0.7859
Pure Error	0.9475	3	0.3158		
Cor Total	2.73	11			

3 Response Surface Methodology

The applied face-centered central composite design (FC-CCD) for an arbitrary three-factor design is shown in Figure S2.

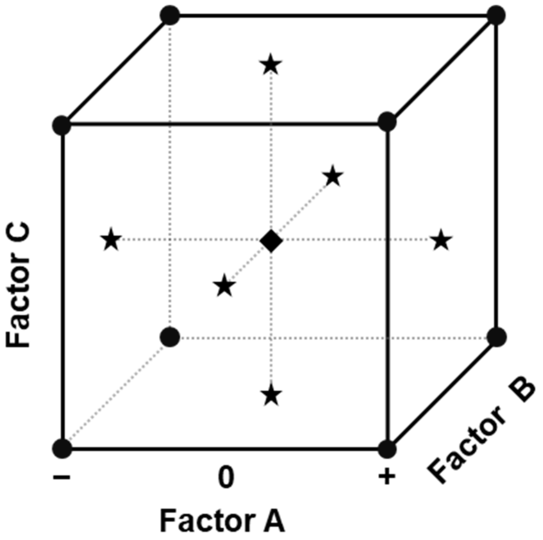

Figure S2: Design geometry of an arbitrary three-factor FC-CCD. Each factor is tested at three levels (−, 0 and +).

The actual FC-CCD applied in this work is illustrated in Table S3. The design consists of a full factorial two-level design ($2^5 = 32$ runs), axial face-centered points (10 runs) as well as eight center points. For purposes of better overview, the runs are listed in standard order. However, in order to avoid lurking time-related errors, the practical order of runs was randomized. It must be stated, that we actively chose a very extensive and comprehensive design in order to provide the best possible prediction accuracy. If financial resources or time are limited, there are various other reasonable designs offering a better suited compromise between prediction accuracy and experimental effort.

Table S3: Standard order experimental design of the FC-CCD. Display of the actual factor values as well as the observed responses.

Run	T / °C	t / min	R_M / a.u.	R_I / a.u.	w_s / a.u.	p^a / %	$\bar{M}_{n,th}{}^a$ / kDa	$Đ^b$ / a.u.	$\bar{M}_{n,app}{}^b$ / kDa
**1**	75	120	200	0.025	10	10.5	2.1	1.29	3.2
**2**	85	120	200	0.025	10	18.2	3.4	1.38	5.4
**3**	75	400	200	0.025	10	36.6	6.4	1.22	3.7
**4**	85	400	200	0.025	10	40.2	7.2	1.45	4.6
**5**	75	120	500	0.025	10	8.4	3.9	1.3	2.2
**6**	85	120	500	0.025	10	12	5	1.45	8.7
**7**	75	400	500	0.025	10	22.2	9.8	1.29	4.6
**8**	85	400	500	0.025	10	26.2	11	1.45	6.0
**9**	75	120	200	0.1	10	25.6	4.7	1.3	6.3
**10**	85	120	200	0.1	10	39.8	7.1	1.22	3.0
**11**	75	400	200	0.1	10	59.9	10.5	1.24	10
**12**	85	400	200	0.1	10	62.6	11	1.33	7.3
**13**	75	120	500	0.1	10	18.5	7.9	1.34	3.4
**14**	85	120	500	0.1	10	26	10.9	1.28	3.8
**15**	75	400	500	0.1	10	43.4	19.6	1.24	6.5
**16**	85	400	500	0.1	10	44.7	19.3	1.35	4.2
**17**	75	120	200	0.025	20	19.1	3.6	1.21	6.4
**18**	85	120	200	0.025	20	24.8	4.5	1.22	6.1
**19**	75	400	200	0.025	20	48.3	8.5	1.23	3.8
**20**	85	400	200	0.025	20	52.6	9.2	1.36	5.1
**21**	75	120	500	0.025	20	8.4	3.9	1.25	5.7
**22**	85	120	500	0.025	20	17.3	7.7	1.26	5.2
**23**	75	400	500	0.025	20	36.8	16	1.25	3.4
**24**	85	400	500	0.025	20	36.5	15.8	1.35	5.4
**25**	75	120	200	0.1	20	31.2	5.6	1.16	6.4
**26**	85	120	200	0.1	20	51.5	9.1	1.23	8.4
**27**	75	400	200	0.1	20	75.6	13.2	1.25	3.4
**28**	85	400	200	0.1	20	74	12.8	1.33	7.3
**29**	75	120	500	0.1	20	22	9.7	1.19	4.8
**30**	85	120	500	0.1	20	36.1	15.7	1.25	6.3
**31**	75	400	500	0.1	20	55.1	23.8	1.23	5.0
**32**	85	400	500	0.1	20	57.1	25.1	1.38	2.3
**33**	75	260	350	0.0625	15	36.4	11	1.21	7.7
**34**	85	260	350	0.0625	15	43.8	13.4	1.27	4.2
**35**	80	120	350	0.0625	15	23.8	7.4	1.25	5.3
**36**	80	400	350	0.0625	15	52	15.8	1.29	2.8
**37**	80	260	200	0.0625	15	52.2	9.2	1.24	6.8
**38**	80	260	500	0.0625	15	37.4	16.2	1.26	6.3
**39**	80	260	350	0.025	15	28.8	9.2	1.27	7.6
**40**	80	260	350	0.1	15	49.8	15.2	1.25	9.5
**41**	80	260	350	0.0625	10	36.7	11.2	1.27	8.4
**42**	80	260	350	0.0625	20	47.6	14.3	1.24	4.9
**43**	80	260	350	0.0625	15	40	12.2	1.26	5.0

Run	T / °C	t / min	R_M / a.u.	R_I / a.u.	w_s / a.u.	p^a / %	$\bar{M}_{n,th}{}^a$ / kDa	$Đ^b$ / a.u.	$\bar{M}_{n,app}{}^b$ / kDa
**44**	80	260	350	0.0625	15	42.7	13	1.31	4.5
**45**	80	260	350	0.0625	15	43	13.1	1.26	4.4
**46**	80	260	350	0.0625	15	41.7	12.7	1.25	5.6
**47**	80	260	350	0.0625	15	42.7	12.8	1.28	2.5
**48**	80	260	350	0.0625	15	41.8	12.8	1.24	6.3
**49**	80	260	350	0.0625	15	44	13.2	1.27	7.3
**50**	80	260	350	0.0625	15	42.9	12.8	1.25	6.2

[a] Determined via ^{1}H NMR spectroscopy and referencing to DMF. [b]Measured by SEC at 50 °C in an 0.1 M $NaNO_3$ aqueous (Milli-Q® quality) solution with an added 0.05wt% NaN_3 and calibration with polyethylene glycol.

3.1 ANOVA and Diagnostics: Monomer conversion

The ANOVA table for the response p is listed in Table S4.

Table S4: ANOVA table for the response p. The interaction $T\,w_s$ was included into the model in order to maintain model hierarchy.

Source	Sum of Squares	df	Mean Square	F-value	p-value
**Model of *p***	11977.37	18	665.41	242.12	< 0.0001
T	326.74	1	326.74	118.89	< 0.0001
t	5453.42	1	5453.42	1984.33	< 0.0001
R_M	1354.50	1	1354.50	492.86	< 0.0001
R_I	3125.76	1	3125.76	1137.37	< 0.0001
w_s	776.65	1	776.65	282.60	< 0.0001
$T\,t$	136.13	1	136.13	49.53	< 0.0001
$T\,R_I$	16.53	1	16.53	6.02	0.0200
$T\,w_s$	2.42	1	2.42	0.8806	0.3553
$t\,R_M$	97.30	1	97.30	35.40	< 0.0001
$t\,R_I$	52.53	1	52.53	19.11	0.0001
$t\,w_s$	74.42	1	74.42	27.08	< 0.0001
$R_M\,R_I$	37.84	1	37.84	13.77	0.0008
T^2	11.29	1	11.29	4.11	0.0514
t^2	47.90	1	47.90	17.43	0.0002
R_M^2	18.05	1	18.05	6.57	0.0155
R_I^2	21.67	1	21.67	7.88	0.0086
$T\,t\,R_I$	43.71	1	43.71	15.91	0.0004
$T\,t\,w_s$	16.82	1	16.82	6.12	0.0190
**Residual**	85.20	31	2.75		
Lack of Fit	75.26	24	3.14	2.21	0.1416
Pure Error	9.94	7	1.42		
**Cor Total**	12062.56	49			

The coded model of p is displayed in Table S5 along with the goodness-of-fit measures. The model of p has an insignificant lack of fit (p-value > 0.05), explains more than 99% of the variation and does not contain any irrelevant model terms.

Table S5: Coded prediction model of p as well as the goodness-of-fit measures.

**Model of p**		***Goodness-of-Fit Measures***	
Coefficient	***Term***		
42.3		R^2	0.9929
+ 3.10	T	Adjusted R^2	0.9888
+ 12.7	t	Predicted R^2	0.9780
− 6.31	R_M		
+ 9.59	R_I		
+ 4.78	w_s		
− 2.06	$T\,t$		
+ 0.719	$T\,R_I$		
+ 0.275	$T\,w_s$		
− 1.74	$t\,R_M$		
+ 1.28	$t\,R_I$		
+ 1.52	$t\,w_s$		
− 1.09	$R_M\,R_I$		
− 2.08	T^2		
− 4.28	t^2		
+ 2.62	R_M^2		
− 2.88	R_I^2		
− 1.17	$T\,t\,R_I$		
− 0.725	$T\,t\,w_s$		

Whenever a new model is computed by regression, it is mandatory to perform model diagnostics in order to check the Gauß-Markow assumptions. The diagnostics will uncover potential outliers as well as the potential necessity for data transformation (to uncover possible assumption violations).

Figure S3 displays a normal plot of residuals (top left), a residuals versus predicted responses plot (top right) and a residuals versus run plot (bottom) for the prediction model of p. Residuals are the deviation of the observed response from the response predicted by the respective model. In order to improve outlier detection, the residuals in the graphs are externally studentized.

As indicated by the normal plot of residuals (top left), the residuals appear to be normally distributed. The residuals versus predicted responses plot (top right) indicates no outlier as all residuals fall within the so-called Bonferroni corrected confidence limits (Cl).[1] These limits are calculated according to equation S3, in which t is a function of the $\alpha/2n$-quantile of a t-distribution with n–1–k degrees of freedom (n is the number of experiments and k the amount of coefficients for the respective model). All CIs in this work stem from an α-value of 0.05:

$$\mathrm{CI} = \pm\, \mathrm{t}\left(\frac{\alpha}{2\,n}, n - 1 - k\right). \tag{S3}$$

Moreover, there is no sign of systematically increasing or decreasing variability implying homoscedasticity. The residuals versus run plot (bottom) shows no sign of an obvious time-lurking effect. The model seems to be well suited for accurate and reliable value prediction.

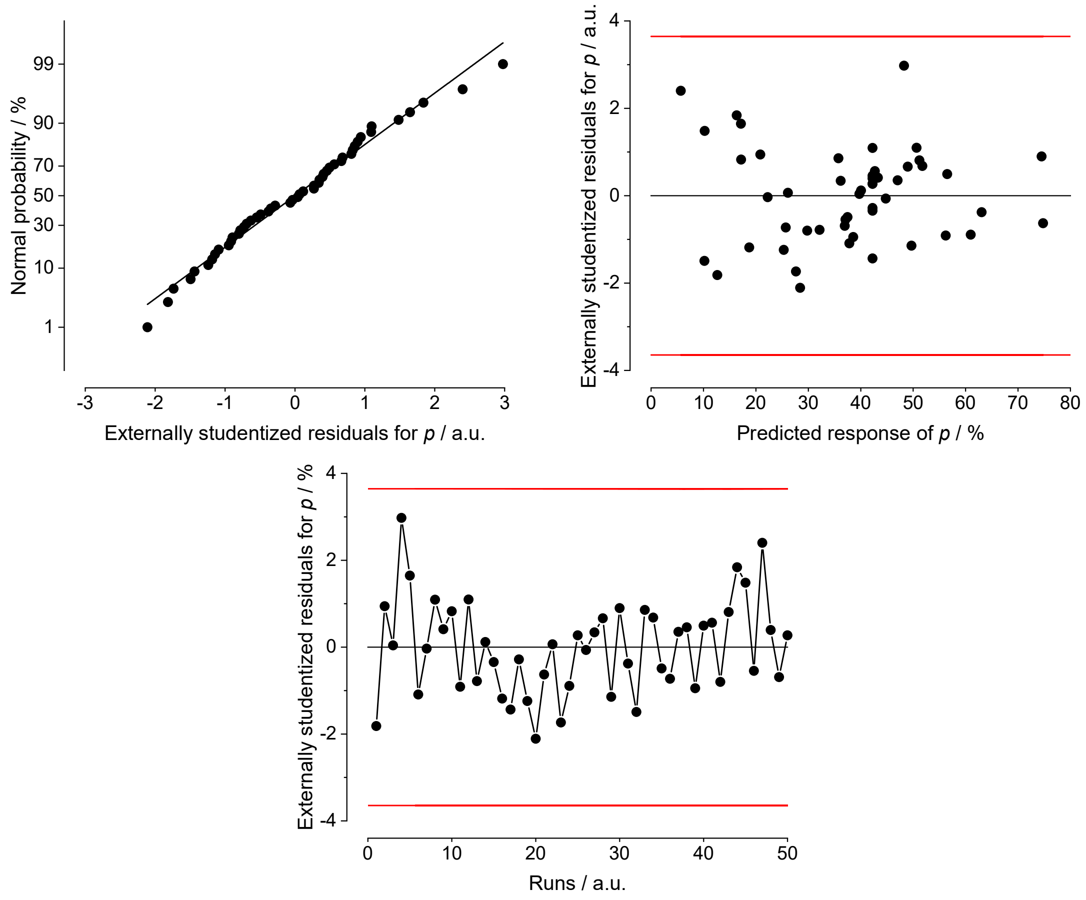

Figure S3: Diagnostic plots for the prediction model of *p*. Top left: Normal probability plot of residuals. Top right: Display of the residuals versus the predicted response. Bottom: Residuals versus run plot. The red lines from the top right and bottom plot are Bonferroni corrected confidence limits ($\alpha = 0.05$).

3.2 ANOVA and Diagnostics: Theoretical Number Average Molecular Weight

The ANOVA table for the response $\overline{M}_{n,th}$ is listed in Table S6.

Table S6: ANOVA table for the response $\overline{M}_{n,th}$. The interaction $T\,w_s$ was included into the model in order to maintain model hierarchy.

Source	Sum of Squares	df	Mean Square	F-value	p-value
**Model of $\overline{M}_{n,th}$**	1219.14	18	67.73	90.64	< 0.0001
T	23.06	1	23.06	30.86	< 0.0001
t	443.52	1	443.52	593.54	< 0.0001
R_M	255.48	1	255.48	341.89	< 0.0001
R_I	259.88	1	259.88	347.78	< 0.0001
w_s	66.36	1	66.36	88.81	< 0.0001
$T\,t$	10.58	1	10.58	14.16	0.0007
$T\,R_I$	1.28	1	1.28	1.71	0.2002
$T\,w_s$	0.9800	1	0.9800	1.31	0.2609
$t\,R_M$	42.78	1	42.78	57.25	< 0.0001
$t\,R_I$	6.85	1	6.85	9.16	0.0049
$t\,w_s$	6.85	1	6.85	9.16	0.0049
$R_M\,R_I$	27.75	1	27.75	37.14	< 0.0001
T^2	1.11	1	1.11	1.48	0.2330
t^2	4.09	1	4.09	5.47	0.0259
R_M^2	0.0586	1	0.0586	0.0784	0.7813
R_I^2	1.11	1	1.11	1.48	0.2330
$T\,t\,R_I$	2.64	1	2.64	3.54	0.0693
$T\,t\,w_s$	1.62	1	1.62	2.17	0.1510
**Residual**	23.16	31	0.7473		
Lack of Fit	22.51	24	0.9379	10.02	0.0021
Pure Error	0.6550	7	0.0936		
**Cor Total**	1242.31	49			

The coded model of $\overline{M}_{n,th}$ is displayed in Table S7 along with the goodness-of-fit measures. The model of $\overline{M}_{n,th}$ has an insignificant lack of fit (p-value > 0.05), explains more than 98% of the variation and does not contain any irrelevant model terms.

Table S7: Coded prediction model of $\overline{M}_{n,th}$ as well as the goodness-of-fit measures.

**Model of $\overline{M}_{n,th}$** *Coefficient*	*Term*	***Goodness-of-Fit Measures***	
12.8		R^2	0.9814
+ 0.824	T	Adjusted R^2	0.9705
+ 3.61	t	Predicted R^2	0.9379
+ 2.74	R_M		
+ 2.76	R_I		
+ 1.40	w_s		
− 0.575	$T\,t$		
+ 0.200	$T\,R_I$		
+ 0.175	$T\,w_s$		
+ 1.16	$t\,R_M$		
+ 0.463	$t\,R_I$		
+ 0.463	$t\,w_s$		
+ 0.931	$R_M\,R_I$		
− 0.650	T^2		
− 1.25	t^2		
− 0.150	R_M^2		
− 0.650	R_I^2		
− 0.288	$T\,t\,R_I$		
− 0.225	$T\,t\,w_s$		

Figure S4 displays a normal plot of residuals (top left), a residuals versus predicted responses plot (top right) and a residuals versus run plot (bottom) for the prediction model of $\overline{M}_{n,th}$. In order to address the differing standard error for each residual, the residuals in the graphs are externally studentized.

The normal plot of residuals (top left) indicates that the residuals are normally distributed. The residuals versus predicted responses plot (top right) indicates no outlier as all residuals fall within the Bonferroni corrected confidence limits. The graph does not show systematically increasing or decreasing variability, again, implying homoscedasticity. Also, the residuals versus run plot (bottom) shows no sign of an obvious time-lurking effect. Like the prediction model of p, the model of $\overline{M}_{n,th}$ also seems to be well suited for accurate and reliable value prediction.

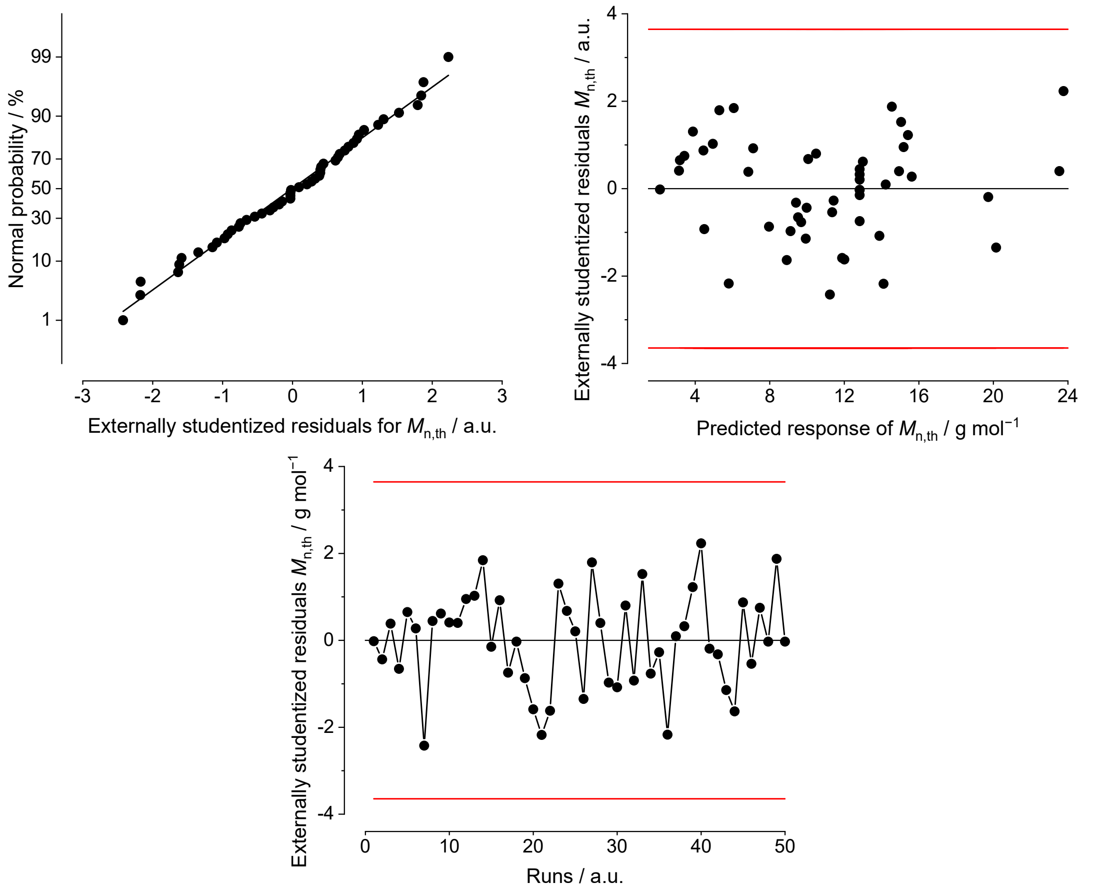

Figure S4: Diagnostic plots for the prediction model of $\overline{M}_{n,th}$. Top left: Normal probability plot of residuals. Top right: Display of the residuals versus the predicted response. Bottom: Residuals versus run plot. The red lines from the top right and bottom plot are Bonferroni corrected confidence limits ($\alpha = 0.05$).

3.3 ANOVA and Diagnostics: Apparent Number Average Molecular Weight

The ANOVA table for the response $\overline{M}_{n,app}$ is listed in Table S8.

Table S8: ANOVA table for the response $\overline{M}_{n,app}$. The interactions $T\ w_s$ and $R_I\ w_s$ were included into the model to maintain model hierarchy.

Source	Sum of Squares	df	Mean Square	F-value	p-value
**Model of $\overline{M}_{n,app}$**	166.10	18	9.23	67.92	< 0.0001
T	2.28	1	2.28	16.76	0.0003
t	53.38	1	53.38	392.83	< 0.0001
R_M	37.91	1	37.91	278.98	< 0.0001
R_I	29.18	1	29.18	214.78	< 0.0001
w_s	8.10	1	8.10	59.65	< 0.0001
$T\ t$	2.15	1	2.15	15.84	0.0004
$T\ R_M$	1.40	1	1.40	10.32	0.0031
$T\ w_s$	0.0903	1	0.0903	0.6647	0.4211
$t\ R_M$	6.57	1	6.57	48.36	< 0.0001
$t\ R_I$	0.3828	1	0.3828	2.82	0.1033
$t\ w_s$	3.32	1	3.32	24.40	< 0.0001
$R_M\ R_I$	2.48	1	2.48	18.22	0.0002
$R_M\ w_s$	2.48	1	2.48	18.22	0.0002
$R_I\ w_s$	0.0703	1	0.0703	0.5175	0.4773
T^2	0.4855	1	0.4855	3.57	0.0681
t^2	2.13	1	2.13	15.67	0.0004
$T\ t\ w_s$	0.5778	1	0.5778	4.25	0.0477
$t\ R_I\ w_s$	2.94	1	2.94	21.64	< 0.0001
**Residuals**	4.21	31	0.1359		
Lack of Fit	4.02	24	0.1674	6.01	0.0103
Pure Error	0.1950	7	0.0279		
**Cor Total**	170.32	49			

The coded model of $\overline{M}_{n,app}$ is displayed in Table S9 along with the goodness-of-fit measures. The model of $\overline{M}_{n,app}$ explains more than 97% of the variation and does not contain any irrelevant model terms.

Table S9: Coded prediction model of $\overline{M}_{n,app}$ as well as the goodness-of-fit measures.

**Model of $\overline{M}_{n,app}$**		***Goodness-of-Fit Measures***	
Coefficient	***Term***		
6.23		R^2	0.9753
+0.259	T	Adjusted R^2	0.9609
+1.25	t	Predicted R^2	0.9129
+1.06	R_M		
+0.927	R_I		
+0.488	w_s		
−0.259	$T\,t$		
+0.209	$T\,R_M$		
+0.053	$T\,w_s$		
+0.453	$t\,R_M$		
+0.109	$t\,R_I$		
+0.322	$t\,w_s$		
+0.278	$R_M\,R_I$		
+0.278	$R_M\,w_s$		
+0.047	$R_I\,w_s$		
−0.366	T^2		
−0.766	t^2		
−0.134	$T\,t\,w_s$		
−0.303	$t\,R_I\,w_s$		

Figure S5 shows a normal plot of residuals (top left), a residuals versus predicted responses plot (top right) as well as a residuals versus run plot (bottom) for the prediction model of $\overline{M}_{n,app}$. As the standard error for each residual differs, the residuals in the graphs are externally studentized.

As the residuals show linear behavior in the normal plot of residuals (top left) this indicates that the residuals are normally distributed. The residuals versus predicted responses plot (top right) shows no outlier as all residuals fall within the Bonferroni corrected confidence limits. Additionally, the graph does not show systematically increasing or decreasing variability, which signalizes homoscedasticity. Also, the residuals versus run plot (bottom) shows no sign of an obvious time-lurking effect.

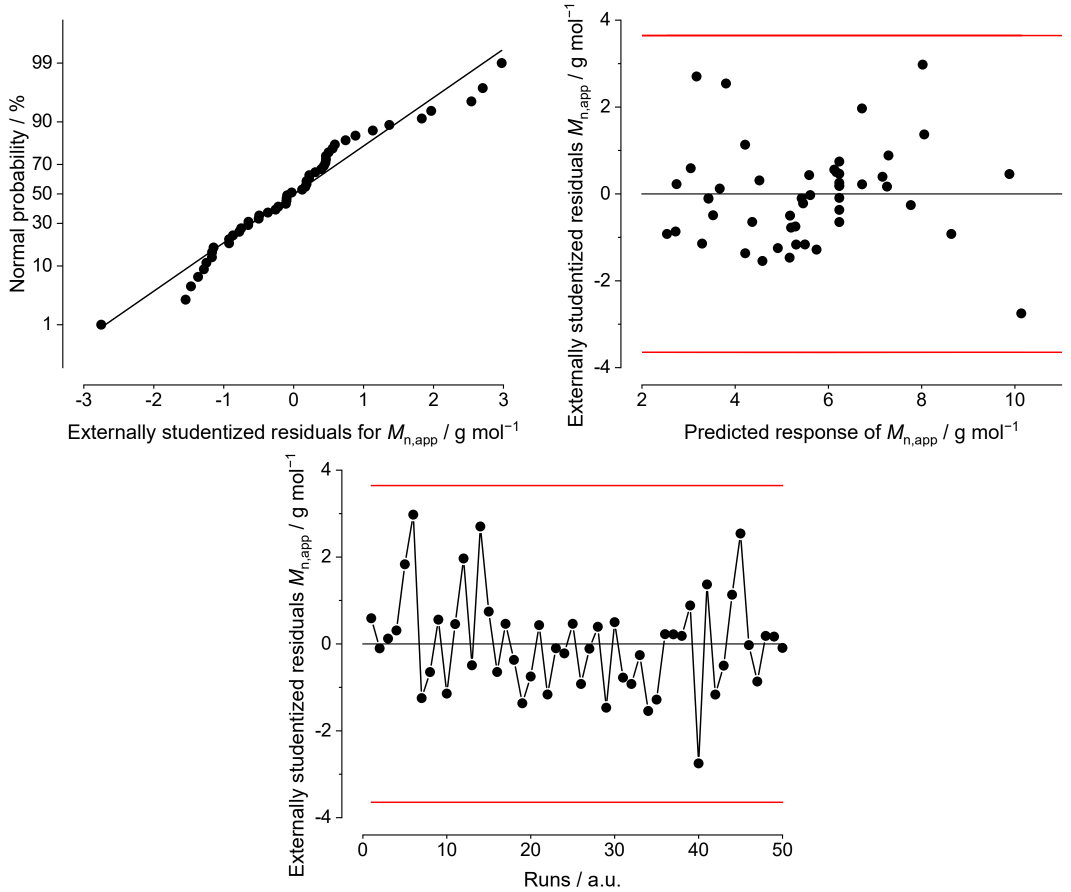

Figure S5: Diagnostic plots for the prediction model of $\overline{M}_{n,app}$. Top left: Normal probability plot of residuals. Top right: Display of the residuals versus the predicted response. Bottom: Residuals versus run plot. The red lines from the top right and bottom plot are Bonferroni corrected confidence limits ($\alpha = 0.05$).

3.4 ANOVA and Diagnostics: Dispersity

The ANOVA table for the response *Đ* is listed in Table S10.

Table S10: ANOVA table for the response *Đ*.

Source	Sum of Squares	df	Mean Square	F-value	p-value
**Model of *Đ***	0.1845	12	0.0154	34.03	< 0.0001
T	0.0544	1	0.0544	120.37	< 0.0001
t	0.0128	1	0.0128	28.35	< 0.0001
R_M	0.0062	1	0.0062	13.77	0.0007
R_I	0.0128	1	0.0128	28.35	< 0.0001
w_s	0.0300	1	0.0300	66.39	< 0.0001
$T\,t$	0.0200	1	0.0200	44.25	< 0.0001
$T\,R_I$	0.0066	1	0.0066	14.63	0.0005
$T\,w_s$	0.0002	1	0.0002	0.4425	0.5100
$t\,w_s$	0.0113	1	0.0113	24.89	< 0.0001
$R_I\,w_s$	0.0055	1	0.0055	12.20	0.0013
t^2	0.0103	1	0.0103	22.69	< 0.0001
$T\,R_I\,w_s$	0.0144	1	0.0144	31.97	< 0.0001
**Residual**	0.0167	37	0.0005		
Lack of Fit	0.0133	30	0.0004	0.9142	0.6080
Pure Error	0.0034	7	0.0005		
**Cor Total**	0.2013	49			

The coded model of *Đ* is displayed in Table S11 along with the goodness-of-fit measures. The model of *p* has an insignificant lack of fit (p-value > 0.05), explains more than 98% of the variation and does not contain any irrelevant model terms.

Table S11: Coded prediction model of *Đ* as well as the goodness-of-fit measures.

**Model of *Đ***		***Goodness-of-Fit Measures***	
Coefficient	***Term***		
1.26		R^2	0.9169
+ 0.0400	T	Adjusted R^2	0.8900
+ 0.0194	t	Predicted R^2	0.8347
+ 0.0135	R_M		
− 0.0194	R_I		
− 0.0297	w_s		
+ 0.0250	$T\,t$		
− 0.0144	$T\,R_I$		
− 0.0025	$T\,w_s$		
+ 0.0188	$t\,w_s$		
+ 0.0131	$R_I\,w_s$		
+ 0.0307	t^2		
+ 0.0212	$T\,R_I\,w_s$		

Figure S6 displays a normal plot of residuals (top left), a residuals versus predicted responses plot (top right) and a residuals versus run plot (bottom) for the prediction model of *Đ*. In order to address the differing standard error for each residual, the residuals in the graphs are externally studentized.

The residuals are in reasonable agreement with the straight line in the normal probability plot (top left) indicating a normal distribution. Furthermore, there is neither a sign of an outlier since the residuals all fall within the Bonferroni corrected confidence limits nor any indication towards systematically increasing or decreasing variability suggesting homoscedasticity. As the residuals versus run plot (bottom) appears to scatter randomly around an externally studentized residual of 0, there is no sign of an obvious time-lurking effect. The prediction model of *Đ* seems to be well suited for accurate and reliable value prediction.

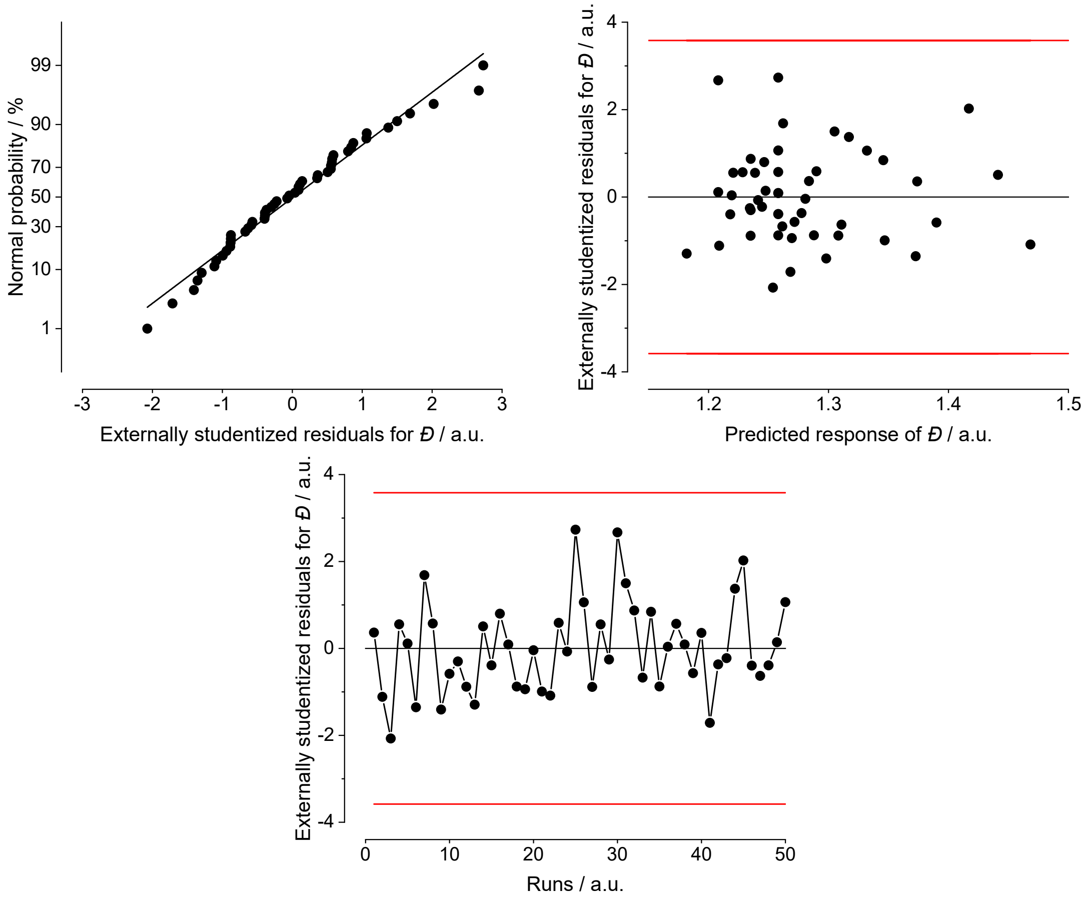

Figure S6: Diagnostic plots for the prediction model of *Đ*. Top left: Normal probability plot of residuals. Top right: Display of the residuals versus the predicted response. Bottom: Residuals versus run plot. The red lines from the top right and bottom plot are Bonferroni corrected confidence limits ($\alpha = 0.05$).

.

4 Decomposition of ACVA

The activation Energy E_A of the decomposition of racemic 4,4′-Azobis(4-cyanovaleric acid) (ACVA) amounts to 132.2 kJ mol^{-1} and the pre-exponential factor A has a value of $4.76 \cdot 10^{15}$ s^{-1}.[2] According to Arrhenius' law (Equation S4), the decomposition rate coefficient k_d at 85 °C obtains a value of:

$$k_d = A \exp\left(-E_A / RT\right) = 2.489 \cdot 10^{-4} s^{-1}. \quad (S4)$$

After 260 min at 85 °C in aqueous solution, 98% of the initial ACVA concentration $[I]_0$ has decomposed according to Equation S5:

$$1 - [I]/[I]_0 = 1 - \exp(-k_d\, t) = 0.98 \quad (S5)$$

5 Model Validation

Three arbitrary polymerization goals were defined. These goals as well as the respective optimal factor settings obtained from the prediction models are listed in Table S12. Polymerization goals are highlighted in bold letters.

Table S12: Listing of the paradigmatic polymerization goals and their respective optimal factor settings.

Goal	$\bar{M}_{n,th}$[a] / kDa	p[a] / %	$\bar{M}_{n,app}$ / kDa	$Đ$ / a.u.	T / °C	t / min	R_M / a.u.	R_I / a.u.	w_s / a.u.
1	**8.0**	45.2	4.70	**Minimize**	75	186	200	0.1	20
2	**13.1**	57.5	6.35	**1.25**	80	321	256	0.091	13.6
3	**23.0**	58.8	9.87	**1.32**	82	390	466	0.1	19.95

[a] Determined via ^{1}H NMR spectroscopy and referencing to DMF. [b]Measured by SEC at 50 °C in an 0.1 M $NaNO_3$ aqueous (Milli-Q® quality) solution with an added 0.05wt% NaN_3 and calibration with polyethylene glycol.

Comprehensive validation data such as the predicted and observed responses as well as the 95% predictive intervals (PIs) is given in Table S13.

Table S13: Predicted and observed responses as well as the 95% PIs of the three paradigmatic polymerization goals.

Goal	Response	Predicted	95% PI low	95% PI high	Observed
1	p / %	45.2211	41.0956	49.3466	45.7
	$\bar{M}_{n,th}$ / kDa	7.95615	5.80495	10.1073	8.10
	$Đ$ / a.u.	1.16585	1.11668	1.21502	1.17
	$\bar{M}_{n,app}$ / kDa	4.70198	3.78601	5.61796	4.60
2	p / %	57.4723	53.8000	61.1445	56.7
	$\bar{M}_{n,th}$ / kDa	13.0571	11.1422	14.9719	12.6
	$Đ$ / a.u.	1.25242	1.20764	1.29719	1.25
	$\bar{M}_{n,app}$ / kDa	6.3472	5.55299	7.14141	6.40
3	p / %	58.8018	54.7417	62.8618	57.8
	$\bar{M}_{n,th}$ / kDa	23.0066	20.8895	25.1237	22.9
	$Đ$ / a.u.	1.32145	1.27417	1.36874	1.32
	$\bar{M}_{n,app}$ / kDa	9.86659	8.96695	10.7662	10.1

[a] Determined via ^{1}H NMR spectroscopy and referencing to DMF. [b]Measured by SEC at 50 °C in an 0.1 M $NaNO_3$ aqueous (Milli-Q® quality) solution with an added 0.05wt% NaN_3 and calibration with polyethylene glycol.

References

1. Myers, R.; Montgomery, D.; Anderson-Cook, C. *Response Surface Methodology*; Wiley: New Yersey, 2016; ISBN 9781118916018.

2. Zhou, Y.; Zhang, Z.; Postma, A.; Moad, G. Kinetics and mechanism for thermal and photochemical decomposition of 4,4′-azobis(4-cyanopentanoic acid) in aqueous media. *Polym. Chem.* **2019**, *10*, 3284–3287, doi:10.1039/c9py00507b.
